# Detection of Otosclerosis-Specific Measles Virus Receptor (Cd46) Protein Isoforms

**DOI:** 10.1155/2013/479482

**Published:** 2013-06-20

**Authors:** Balázs Liktor, Péter Csomor, Tamás Karosi

**Affiliations:** ^1^Bajcsy-Zsilinszky Hospital, Department of Otolaryngology, Budapest, Hungary; ^2^University of Debrecen, Medical and Health Science Center, Department of Rheumatology, Debrecen, Hungary; ^3^University of Debrecen, Medical and Health Science Center, Department of Otolaryngology and Head and Neck Surgery, Nagyerdei Krt. 98, Debrecen 4032, Hungary

## Abstract

Genetic predisposition of otosclerosis has long been suspected, but unclarified. Unique coexpression pattern of measles virus receptor (CD46) splicing isoforms in the human otic capsule is assumed, since otosclerosis is a measles virus-associated organ-specific disease. In order to identify CD46 involved in the pathogenesis of otosclerosis, we used representative groups of histologically diagnosed otosclerotic, nonotosclerotic, and normal stapes footplates (*n* = 109). Consecutive histopathological examinations and CD46-specific Western blot analysis were performed. Normal and nonotosclerotic stapes footplates showed consistent expression of the conventional *c, d, e, f*, and *l* CD46 isoforms. In contrast, four novel isoforms *(os1–4)* translated as intact proteins were additionally detected in each otosclerotic specimen. The study herein presented provides evidence for the otosclerosis-associated expression pattern of CD46. This finding might explain the organ-specific, virus-associated and autoimmune-inflammatory pathogenesis of otosclerosis. Regarding our current knowledge, this is the first report that confirms the presence of four new disease-specific protein variants of CD46.

## 1. Introduction

Otosclerosis is a complex inflammatory bone remodeling disorder of the human otic capsule that leads to progressive conductive and/or sensorineural hearing loss as a consequence of stapes footplate fixation and cochlear bone resorption with endosteal involvement [1]. In the Caucasian population, the prevalence of clinical otosclerosis is 0.3–0.4% of the general population, 5–9% of those with hearing loss, and 18–22% of those with conductive hearing loss [1, 2]. Silent otosclerotic foci are more common: histological otosclerosis without clinical symptoms has been reported as 8–11% in large unselected autopsy series [2]. Otosclerotic foci are limited to the temporal bone, and no lesions have been found outside of the ear [2–5]. Otosclerosis takes approximately two thirdS of stapes ankylosis cases leading to consecutive conductive hearing loss [6]. Differential diagnosis is still based on postoperative histological analysis of the removed stapes footplates [3, 4, 7].

However, several hypotheses suggest viral, autoimmune, and endocrine factors in the genesis of disease, etiopathogenesis of otosclerosis remained unexplained [3, 8–10]. Genetic factors must play a significant role in the etiopathogenesis of otosclerosis, although the precise mode of inheritance is still uncertain [3, 5, 10]. The potential etiologic role of measles virus in the pathogenesis of otosclerosis was suggested in the past twenty-five years [8, 9]. The otosclerotic otic capsule is assumed to exhibit a unique paramyxovirus favoring receptor expression pattern, which could be the basis of the pathogenesis of otosclerosis and also the reduced humoral immune response against measles virus-derived antigens [3, 6, 7]. 

One of the human cellular receptors for measles virus is the CD46 molecule, also known as membrane cofactor protein (MCP) [11]. CD46 has a cofactor activity for inactivation of complement components C3b and C4b by serum factor I, which protects the host cell from damage by the complement system [11]. Signals mediated by CD46 have a great influence on T-cell activation [11, 12]. Beyond these functions, CD46 plays a role in the pathogenesis of various inflammatory disorders. Its therapeutic potential in inflammatory diseases has also been suggested. Elevated serum levels of CD46 have been reported in systemic lupus erythematosus (SLE) [13]. Recombinant soluble CD46 has been introduced to animal models of various inflammatory diseases [14]. For example, CD46 treatment inhibited acute cardiac transplant rejection [14]. Accordingly, targeted therapies using recombinant CD46 may be useful in autoimmune-inflammatory conditions [3, 14]. The mRNA of CD46 is translated from a single gene linked to chromosome 1q32; however, it is posttranslationally modified by alternative splicing resulting in 14 known splicing variants and corresponding protein isoforms [6, 11, 12]. Different numbers of CD46 isoforms are coexpressed by all nucleated human cells in various patterns [11]. However, specific functions have not been associated to isoform coexpression yet [6, 11]. In 2008, Karosi et al. described four novel otosclerosis-associated splicing variants of CD46 mRNA; however, no additional reports have arisen about the translated and corresponding protein isoforms associated to the etiopathogenesis of otosclerosis [6]. 

The present study investigates the coexpression pattern of CD46 protein isoforms in histologically proven otosclerotic, nonotosclerotic, and normal stapes footplates in order to establish organ-specific, otosclerosis-associated alternative splicing of the measles virus receptor CD46.

## 2. Materials and Methods

### 2.1. Patients and Controls

Altogether 109 stapes footplates (male = 39, female = 70) were analyzed. Out of these samples, 92 were ankylotic and removed by stapedectomy and were immediately fixed in 10% (w/v) formaldehyde. The mean age of patients was 41.7 years (range: 21–72 years). Stapes footplate specimens were selected histologically from a larger pool (*n* = 419) of ankylotic stapes samples with the aim of obtaining representative groups of otosclerotic and nonotosclerotic stapes footplates. Partially removed stapes footplates were not included in the study because the anterior or posterior poles containing the bone lesions fixing the stapes were retained in the oval window niche. However, fragmented and reconstructed footplates were not excluded. The diagnosis of ossicular chain fixation was based on clinical, audiometric, and tympanometric findings. Air-bone gap at 1000 Hz was at least 30 dB. Preoperative tympanometry revealed type-As tympanograms in 67.4% and type-A tympanograms in 32.6% of stapes fixation cases. High resolution CT scan was performed in 18 cases that revealed thickening of the stapes footplate in 11 cases with no apparent signs of hypodensity due to otosclerotic foci in the otic capsule or in the stapes footplate. Seventeen cadaver stapes specimens with negative otopathological history were removed by dissection of temporal bones and were employed as negative controls (*n* = 17, male = 8, female = 9). These were removed within 20 hours after death. The mean age of negative controls was 53.5 years (range: 49–69 years). Stapes footplates were collected between January 2008 and March 2009 at the Universities of Debrecen, Pécs and Szeged, and at the Department of Otolaryngology, Bajcsy-Zsilinszky Hospital, Budapest, Hungary. We obtained Hungarian Scientific Research Ethical Committee (ETT-TUKEB/2008) and Institutional Ethical Committee (DE OEC-EB/2008/12) approvals. The study was carried out according to the Declaration of Helsinki.

### 2.2. Histopathological Analysis

A total of 109 stapes footplate specimens were fixed in 10% (w/v) formaldehyde and decalcified in 0.5 M Na-EDTA containing 0.02% (w/v) sodium azide (72 h, 4°C). Specimens were embedded in 15% (w/v) purified gelatin (24 h, 56°C) and refixed in 4% (w/v) paraformaldehyde (24 h, 20°C). Blocks were cryoprotected in 20% (w/v) saccharose solution (2 h, 4°C) and sectioned into 10 *μ*m slides at −25°C (MNT-200, Slee, Mainz, Germany). Slides were stored in 0.1 M PBS containing 0.03% (w/v) sodium azide at 4°C. Two consecutive 10 *μ*m frozen cut “swimming” sections were examined as follows: (1) conventional staining with hematoxylin and eosin (H&E); (2) pooling and CD46-specific Western blot analysis. Active and inactive otosclerosis cases were differentiated by phase-contrast microscopy. Histological examinations were blinded for two independent researchers: Cs.P. analyzed the sections stained by H&E, while T.K. reviewed the preliminary histopathological diagnosis.

### 2.3. CD46-Specific Western Blot

Second series of the histologically analyzed stapes footplates (*n* = 109) was recruited into three pooled groups: otosclerosis (*n* = 50), nonotosclerotic stapes fixation (*n* = 42), and normal stapes footplates (*n* = 17). Pooled specimens were rinsed three times with sterile PBS buffer to remove contaminants and then ground into powder in liquid nitrogen. Samples were lysed in RIPA buffer (150 mM NaCl, and 1% [w/v] NP40, 50 mM Tris-HCl, 5% [w/v] Na-deoxycholate, 0.1% [w/v] SDS, [w/v] 0.01% sodium-azide, 1 mM EDTA) supplemented with Complete EDTA-free Protease Inhibitor Cocktail (Roche, Basel, Switzerland). The protein concentration was determined using Bradford assay. Total protein extracts (10–15 *μ*g) were boiled in SDS loading buffer and separated in a 7% (w/v) SDS-polyacrylamide gel electrophoresis (SDS-PAGE) and electroblotted onto a nitrocellulose membrane. Membrane was blocked in 5% (w/v) skim milk powder in Tris-buffered saline Tween (TBST: 10 mM Tris, 150 mM NaCl, 0.05% [w/v] Tween20; pH = 7.9), and proteins were detected with anti-CD46 (1 : 200; R&D Systems, Minneapolis, MN, USA) and anti-*β*-actin (1 : 1000; Sigma-Aldrich, Deisenhofen, Germany) antibodies in 5% (w/v) skim milk powder in TBST. The blot was washed three times with TBST, incubated with horseradish peroxidase (HRP)-labeled secondary donkey anti-goat (R&D Systems, Minneapolis, MN, USA) and goat anti-rabbit (Santa Cruz, Santa Cruz, CA, USA) antibody at a dilution of 1 : 1000, washed and visualized with Super Signal West Pico Chemiluminescent Substrate (Pierce, Rockford, IL, USA), and exposed to X-ray film (Kodak, London, UK). THP-1, C33A, and HeLa cell lines were used as positive controls of CD46-specific western blot.

## 3. Results

**Figure 1:**
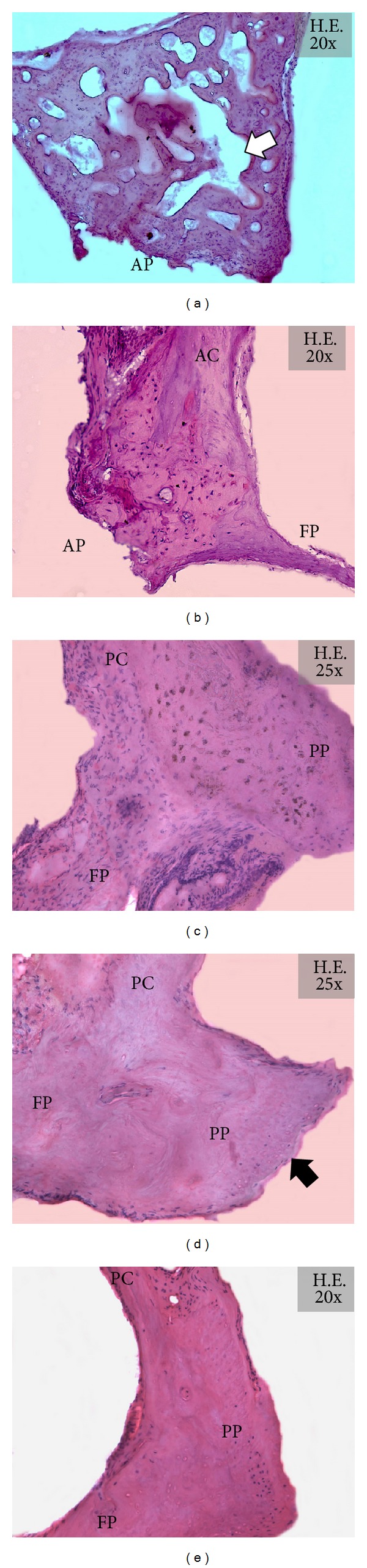
Representative histological findings of ankylotic and normal stapes footplates (H&E). (a) Obliterative otosclerosis with high activity at the anterior pole of the stapes footplate. Hypercellularity and increased numbers of osteoclasts should be noted. “Swiss cheese” pattern of pseudovascular spaces is marked by the white arrow. (b) Inactive otosclerotic focus at the anterior pole of the stapes footplate. Hypocellularity, hypovascularization, marked basophilia, and woven pattern of cement lines can be considered. (c) Hemosiderosis with pathological bone remodeling and active eosinophilic staining. The osteocytes of the stapes footplate are absent and are replaced by hemosiderophages containing dark hemosiderin granules. (d) Annular calcification with decreased cellularity and vascularization. The stapes footplate shows a marked thickening and an angled, bulky posterior pole (black arrow). (e) Histological representation of a normal stapes. Abbreviations: AP: anterior pole; PP: posterior pole; AC: anterior crus; PC: posterior crus; FP: footplate.

Histological diagnosis of otosclerosis was established in 50 ankylotic stapes footplates (Figure 1). According to phase contrast microscopic analysis, otosclerotic foci were seen to be active in 37 stapes footplates and inactive in 13 cases (Figure 1). Active otosclerosis was featured by thickened, and distorted stapes footplate with irregular, woven pattern of cement lines. The focus of otosclerosis was basophilic, hypervascularized and filled with numerous multinucleated osteoclasts, hypercellular fibrous stroma and plump, and distorted osteoblasts (Figure 1). Inactive otosclerosis was characterized by predominantly eosinophilic, woven bone containing some lamellar structure. Osteoclasts and osteoblasts were vanished; vascular spaces were eventually obliterated (Figure 1). In the 42 nonotosclerotic stapes footplates, histological examinations revealed annular calcification with eosinophilic extracellular matrix, decreased cellularity and hypovascularization (*n* = 39), and hemosiderosis with intense bone remodeling of the stapes footplate (*n* = 3) (Figure 1). 

**Figure 2:**
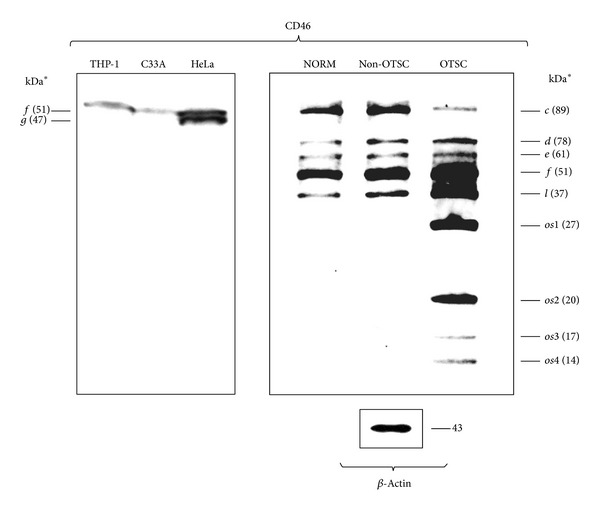
Western blot analysis of CD46 expression pattern in stapes footplate pools with different histopathologies. As controls of CD46 expression and western blot procedure, THP-1, C33A, and HeLa cell lines are characterized by exclusive expression of variant *f* (THP-1, C33A) and coexpression of variants *f *and *g *(HeLa), respectively. Beta-actin was used as the housekeeping control of CD46 coexpression in each stapes footplate pools. At the final level of protein expression, normal, and nonotosclerotic stapes footplates are featured by coexpression of *c, d, e, f*, and *l* isoforms. In contrast, histologically otosclerotic stapes footplates express further four novel protein isoforms of CD46 (*os1–4*). Exact molecular weights of *os* isoforms were calculated by ProteinCalculator (v3.3) online software (http://www.scripps.edu/~cdputnam/protcalc.html).

Conventionally translated isoforms [*c *(89 kDa), *d *(78 kDa), *e *(61 kDa), *f *(51 kDa), and *l* (37 kDa)] of CD46 were detected by western blot analysis of normal and nonotosclerotic stapes footplates (*n* = 59) (Figure 2). As compared to these specimens, in otosclerosis (*n* = 50), four additional protein bands of CD46 isoforms were identified: *os1*, *os2*, *os3*, and *os4* (27 kDa, 20 kDa, 17 kDa, and 14 kDa, resp.) (Figure 2). The estimated molecular weights of *os1–4* isoforms correlated with the mRNA sequence of otosclerosis-associated CD46 splicing variants reported earlier [6]. Coexpression of novel *os1*, *os2*, *os3,* and *os4* CD46 protein isoforms was exclusively associated to the histological diagnosis of otosclerosis in the ankylotic stapes footplates.

## 4. Discussion

 Genetic predisposition for otosclerosis has long been disputed over the last decades, without obvious target genes or mutations to show up [3, 10, 15]. Several studies have reported genetic associations in populations with clinical otosclerosis without histopathological confirmation and have extrapolated the observations to otosclerosis irrespective nonotosclerotic fixations [10, 15]. The majority of genetic studies on families with stapes fixation and on large unselected populations has suggested an autosomal dominant mode of inheritance with incomplete penetrance of approximately 40–45% [10, 15]. Genetic linkage studies have demonstrated the presence of eight loci (OTSC1-8) located on chromosomes 15q, 7q, 6p, 16q, 3q, 6q, and 9p, respectively [3, 10, 15]. Although these loci have been mapped, no causative genes and proteins have been identified and we have a little idea of the molecular process involved in this disease [3, 10, 15]. It has been reported that *COL1A1, BMP2, BMP4, TGFB1, *and* RELN *genes may also contribute to the development of otosclerosis [16–19]. Furthermore, prior associations with these genes account for only a small fraction of the relative risk for otosclerosis or other types of stapes fixation [16–19]. These associations reported earlier cannot explain female dominancy, adult onset, organ specificity, and the inflammatory bone remodeling disorder, all characteristic features of otosclerosis [3, 4]. As to previous results, average life span of an active otosclerotic focus is about 5 to 7 years until inactivation; hence a dynamic genetic hypothesis is necessary to explain this “healing” process [20]. The involvement of T-cells, inflammatory cytokines, and other mediators in otosclerosis suggest an autoimmune-inflammatory nature of the disease [3, 20].

No animal model exists for the otosclerotic bone remodeling disorder, which is associated to the presence of measles virus in the foci [3, 4]. Measles virus is found exclusively in otosclerotic bone in human [8, 9]. This called the attention to the CD46 molecule, which is not expressed as virus receptor in other mammalian species, except humans [6, 11, 12]. Specific diseases have not yet been attributed to CD46 isoform coexpression; however, present study supplies essential information about novel translated isoforms of CD46 emphasizing a potential association between isoform coexpression and otosclerosis. 

**Figure 3:**
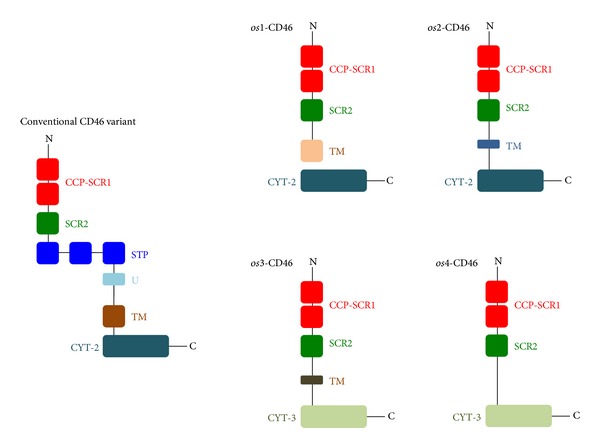
Schematic structure of a conventional and the four otosclerosis-associated CD46 protein variants. Abbreviations: N-N: terminal of CD46; C-C: terminal of CD46; CCP: complement control protein region; SCR-1 and SCR-2: short consensus repeats 1 and 2; STP-Serine: threonine-proline rich region; U: region of unknown significance; TM: transmembrane domain; CYT2 and CYT3: cytoplasmic domain 2 and 3.

Various expressions of different splicing variants of CD46 seem to be one of the acceptable explanations for the genetic background of otosclerosis [6]. Newly described CD46 protein isoforms have a shorter or missing transmembrane and uncommon cytoplasmic domains; however, virus-binding domain remains conservative (Figure 3). Transmembrane and soluble isoforms of CD46 protein have been identified in humans and transgenic mice [6, 11, 12]. In mice, there is an exon spliced alternatively, which allows the encoding of a soluble, cytoplasmic isoform [6, 11]. Although soluble isoforms of human CD46 have been found in different body fluids, mRNA encoding soluble forms remain unidentified until now [11]. The present study supplies direct evidence of a human soluble CD46 isoform *(os4)* produced by alternative splicing (Figures 2-3). These changes should result in functional consequences of signaling that may be responsible for the persisting replication of measles virus. 

Decreased serum level of anti-measles IgG is characteristic for otosclerosis, which is independent from vaccination or measles virus infection and can be determined by expression of otosclerosis-associated CD46 isoforms [6, 7]. Newly described isoforms are supposed to allow virus internalization without TCR-dependent activation of primary CD4 positive T-helper cells and consecutive induction of B-cell-dependent immunoglobulin production [6, 7, 11, 12]. It is supposed that the most important factor in the pathogenesis of otosclerosis is a consecutive autoimmune reaction due to continuous CD46-presented viral antigen stimulation of natural killer cells and CD8 positive T-lymphocytes [6, 11, 12]. Several inflammatory cytokines (TNF-alpha, IL-1, and TGF-beta) and bone-specific proteins (osteoprotegerin, BMP) also may play a secondary promoting role in this process [4, 17, 18, 20].

Proteins expressed as different splicing isoforms are able to imitate regular Mendelian inheritance [3, 6, 10, 11]. Familial cases of otosclerosis showing obscure autosomal dominant inheritance with incomplete penetrance might be considered as a common disease caused by unique alternative splicing of measles virus receptors in the otic capsule [10, 15]. An unresolved question is whether measles virus can induce the expression of new CD46 splicing variants, or existing novel isoforms lead to the increased virus affinity and smooth virus replication [6, 11, 12]? The answer may hide in the expression of different regulatory proteins of alternative splicing leading to a special expression pattern and altered functions of CD46 that could explain the organ-specific and virus-associated pathogenesis of otosclerosis [4, 6, 11, 12]. 

In conclusion, we found a strong association between the expression of novel CD46 protein isoforms and histologically confirmed otosclerosis. Our results are consistent with four new splicing variants of CD46 mRNA being causally related to the viral pathogenesis of otosclerosis [6]. Special expression pattern of CD46 isoforms due to organ-specific alternative splicing may explain genetically determined susceptibility for persisting measles virus infection observed in otosclerosis. In contrast to these observations, in the future, further molecular biological research will be necessary to confirm or exclude our results in the genetic predisposition of otosclerotic bone remodeling disorder.
